# sTWEAK is a leukoaraiosis biomarker associated with neurovascular angiopathy

**DOI:** 10.1002/acn3.51502

**Published:** 2022-01-21

**Authors:** Andrés da Silva‐Candal, Antia Custodia, Iria López‐Dequidt, Manuel Rodríguez‐Yáñez, Maria Luz Alonso‐Alonso, Paulo Ávila‐Gómez, José M. Pumar, José Castillo, Tomás Sobrino, Francisco Campos, Ramón Iglesias‐Rey, Pablo Hervella

**Affiliations:** ^1^ Clinical Neurosciences Research Laboratories (LINC) Health Research Institute of Santiago de Compostela (IDIS) Santiago de Compostela Spain; ^2^ Neurovascular Diseases Laboratory Neurology Service University Hospital Complex of A Coruña Biomedical Research Institute (INIBIC) A Coruña Spain; ^3^ Stroke Unit Department of Neurology Hospital Clínico Universitario Santiago de Compostela Spain; ^4^ Department of Neuroradiology Hospital Clínico Universitario Health Research Institute of Santiago de Compostela (IDIS) Santiago de Compostela Spain

## Abstract

**Objective:**

Leukoaraiosis (LA) refers to white matter lesions of undetermined etiology associated with the appearance and worsening of vascular pathologies. The aim is to confirm an increased frequency and intensity of LA in symptomatic patients with neurovascular pathology compared with asymptomatic subjects, and its association with circulating serum levels of soluble tumor necrosis factor‐like weak inducer of apoptosis (sTWEAK).

**Methods:**

An observational study was conducted in which two groups of patients were compared. Group I (*N* = 242) comprised of asymptomatic subjects with arterial hypertension and/or diabetes or with a history of transient ischemic attacks, and Group II (N = 382) comprised patients with lacunar stroke or deep hemispheric intracerebral hemorrhage (ICH) of hypertensive origin. Serum levels of sTWEAK were analyzed and correlated with prevalence and intensity of LA according to the Fazekas scale.

**Results:**

The prevalence of LA was higher in symptomatic (85.1%) versus asymptomatic patients (62.0%). Logistic regression model showed a significant relation of LA with neurovascular pathologies (OR: 2.69, IC 95%: 1.10–6.59, *p* = 0.003). When stratified according to the Fazekas scale, LA of grade II (OR: 3.53, IC 95%: 1.10–6.59, *p* = 0.003) and specially grade III (OR: 4.66, 95% CI: 1.09–19.84, *p* = 0.037) showed correlation with neurovascular pathologies. Increased sTWEAK levels were found in the symptomatic group in all LA grades (*p* < 0.0001), and associated with 5.06 times more risk of presenting clinical symptoms (OR: 5.06, 95% CI: 2.66–9.75, *p* < 0.0001).

**Interpretation:**

LA showed a higher prevalence in patients with symptomatic lacunar stroke or deep hemispheric ICH. There is an association between sTWEAK levels and LA degree.

## Introduction

Leukoaraiosis (LA) is a radiological term referring to lesions in the white substance through imaging techniques, such as low density on computed tomography (CT), hyperintensity on T2‐weighted or FLAIR images in magnetic resonance image (MRI).^1^, ^2^ Asymptomatic LA is closely linked to a pathological evolution to cerebral small vessel disease (cSVD)^2^ which, in turn, is the prelude of more severe neurological diseases such as early dementia and Alzheimer's disease,^3^, ^4^ lacunar infarcts, or ischemic^5^, ^6^, ^7^, ^8^, ^9^, ^10^ and hemorrhagic stroke.^11^ Interest in LA has grown due to its usefulness as a stroke risk marker,^12^ outcome predictor^9^, ^13^, ^14^ as well as a marker of hemorrhagic transformation after thrombolysis.^10^, ^15^ LA is also found roughly in 30% of asymptomatic patients aged between 50 and 75 years old.^16^ The exact pathways by which LA influences the appearance and evolution of these pathologies is still an unanswered question, suggesting multiple origins and mechanisms of action.^6^, ^17^, ^18^, ^19^ To date, there is no effective treatment to prevent cSVD progession,^20^ highlighting the necessity to understand LA. In this regard, the elucidation of the triggering molecules involved in its appearance and progression could make LA a therapeutic target on its own, helping not only in the treatment of ongoing related diseases but also to prophylactically treat asymptomatic patients avoiding future neurological conditions. Accordingly, in the present study, we have analyzed the association of clinical variables and a serum protein potentially associated with LA progression and severity. We have focused on the soluble tumor necrosis factor‐like weak inducer of apoptosis (sTWEAK), a cytokine of the TNF superfamily^21^, ^22^, ^23^, ^24^, ^25^, ^26^ related to cardiovascular prognosis and endothelial dysfunction closely linked to LA generation and progression.^27^, ^28^, ^29^

## Methods

### Participants and cohorts

A registry‐based observational study was conducted with a total of 624 patients consecutively and prospectively recruited from our databank, who were admitted to the Stroke Unit of the University Clinical Hospital of Santiago de Compostela (Spain). The recruitment period was from January 2008 through December 2018. Patients with hypertension or diabetes were included consecutively and evaluated on a prospective registry.

Subjects were assigned in two different groups:

1 Group I (*n* = 242): Asymptomatic subjects ≥50 years with history of high blood pressure and/or diabetes of >5 years, without neurological vascular manifestations or cognitive deficit screened by primary care physicians (*n* = 105) and subjects with a history of transient ischemic attacks of the carotid territory, without cardioembolic features (*n* = 137) according to the TOAST classification.
2 Group II (*n* = 382): Symptomatic patients with hemispheric lacunar infarctions (*n* = 195) or deep intracerebral hemorrhages of hypertensive mechanism (*n* = 187).

The study was carried out in accordance with the Declaration of Helsinki of the World Medical Association and approved by the Research Ethics Committee of Santiago (Registration code 2019/616 and 2016/399). Informed consent was obtained from each patient or from their relatives after full explanation of the procedures.

### Neuroimaging

A magnetic resonance study was conducted for all the patients enrolled. Imaging of patients with vascular risk (Group I) was conducted in the first quarter after their enrollment in the study. For symptomatic patients (Group II), neuroimages were acquired at admission to the emergency department by neurologists of the stroke unit blinded to clinical and analytical data. The presence and severity of LA were assessed using the Fazekas scale with a total score from 0 to 6 (grade I, 1–2; grade II, 3–4; and grade III, 5–6).^30^ All neuroimages were supervised by the same neuroradiologist blinded to clinical information.

### Clinical variables and biomarkers

Circulating biomarkers were selected based on existing evidence from experimental and clinical studies.^31^, ^32^, ^33^, ^34^, ^35^ After extraction, serum samples were immediately frozen and stored at −80°C until the assay. Due to the retrospective nature of the study, the measurement of biomarkers was not performed simultaneously in all cohorts, but it was performed with the same analytical methods and supervised by the same researchers. Blood samples of vascular risk patients (Group I) were collected at the time of neuroimaging acquisition. For the remaining groups, blood samples were collected on admission. sTWEAK was measured using Human TWEAK ELISA Kit (Elabscience). Minimum assay sensitivity was 1.6 and 4.69 pg/ml, respectively, with an intra‐ and interassay coefficient of variation (CV) <10%. Each sample was assayed in duplicate.

Measurements were performed in an independent laboratory blinded to clinical data.

### Statistical analysis

Initially, a descriptive analysis of the sample was performed. Categorical variables were described as frequency and percentage, and the continuous variables as mean/standard deviation or median/interquartile range, according to their adjustment to a normal distribution (determined by the Kolmogorov–Smirnov test with the Lilliefors corrections). The statistical inference was carried out with the chi‐squared test, ANOVA, or Mann–Whitney test according to the nature of the contrast variable and its adjustment to normality. Data comparison was performed by means of Pearson test for categorical variables and Spearman's rho test for the continuous ones. Finally, a multivariable logistic regression was carried out adjusting for the significant variables found in the previous analysis. ORs and their 95% confidence intervals were calculated. All analyses were performed with IBM SPSS Statistics v.20. Statistical significance was set at *p*‐values <0.05.

The number of patients was estimated to achieve a power of 80% to detect differences in the null hypothesis H_0_ (the prevalence of LA of the two groups is similar) with a level of significance of 5%, assuming an expected LA prevalence of 70% in the asymptomatic group and 85% in the symptomatic group. A total of at least 203 subjects/patients should be enrolled in each cohort, which implies a minimum total sample of 406 patients distributed between the two balanced groups. The calculation was performed with Ene 3.0 program.

### Data availability statement

All data supporting the findings of this study are available to any qualified investigator from the corresponding author upon request.

## Results

### Sample Description

A total of 624 subjects, divided into asymptomatic Group I (*N* = 242) and symptomatic Group II (*n* = 382) were enrolled in this study. An initial descriptive analysis of the cohorts was performed to evaluate differences between groups (Table S1). Demographic variables showed statistical differences in the age of patients (70.1 ± 9.6 vs. 71.5 ± 12.7, *p* = 0.017) and in the previous Rankin scale (0 [0, 0] vs 0 [0, 1], *p* < 0.0001). Symptomatic patients exhibited a higher percentage of ischemic heart disease (2.4% vs 10.3%, *p* = 0.027) and previous symptomatic stroke (0% vs 7.1% p = 0.019). When analyzing molecular variables, clinical inflammation markers such as leukocytes (7.3 ± 1.9 vs. 8.8 ± 2.9 × 10^3^/mmc) and C‐reactive protein (1.29 ± 1.87 vs 4.51 ± 5.17 mg/L) were higher in the symptomatic group (*p* < 0.0001 for both variables). A similar association was found in microalbuminuria (3.4 ± 7.2 vs 13.3 ± 18.1 mg/24 h, *p* < 0.0001), probably due the pathological vascular condition of the patients.

### LA and neurovascular pathology association

The descriptive bivariate study of neuroimaging variables showed an increased number of patients with LA in the symptomatic group compared to the asymptomatic group (85.1% vs 62.0%, *p* < 0.0001). We found statistical differences between groups when the sample was stratified according to the degree of LA (*p* < 0.0001) (Table S1). An adjusted multivariable logistic regression model analysis was performed in order to determine those factors associated with symptomatic lacunar stroke or deep hemispheric ICH (Table 1). Once adjusted for confounding factors, the presence of LA remained the only independent variable correlated with symptomatic lacunar stroke or deep hemispheric ICH (OR: 2.69, 95% CI: 1.10–6.59, *p* = 0.003). As this study was mainly focused on LA, the regression model was repeated including LA stratification from grade I to III. As highlighted in Table 2, LA of grade II (OR: 3.53, 95% CI: 1.10–6.59, *p* = 0.003) and especially grade III (OR: 4.66, 95% CI: 1.09–19.84, *p* = 0.037) correlated with suffering a symptomatic lacunar stroke or deep hemispheric ICH. In this regard, it is particularly important to remark that LA grade I lost this correlation in the adjusted model (OR: 1.67, 95% CI: 0.59–4.79, p = 0.335).

**Table 1:** Multivariable logistic regression model of significant variables using symptomatic patient belonging as dependent variable

Dependent variable: Symptomatic	OR	Nonadjusted	*p*	OR	Adjusted	*p*
Independent variable		95% CI			95% CI	
Age (years)	1.01	1.00–1.02	0.013	0.98	0.95–1.02	0.251
Previous Rankin scale	2.43	1.59–3.81	<0.0001	1.06	0.54–2.08	0.871
Ischemic heart disease	0.47	0.25–0.91	0.025	0.30	0.07–1.21	0.091
Previous stroke	1.18	1.02–1.96	0.005	1.09	0.65–1.98	0.089
Leukocytes × 10^3^/mmc	1.29	1.19–1.39	<0.0001	1.04	0.84–1.23	0.736
C‐reactive protein (mg/L)	1.40	1.23–1.59	<0.0001	1.22	0.99–1.50	0.056
Microalbuminuria (mg/24 h)	1.08	1.03–1.13	0.001	1.06	0.99–1.40	0.074
Leukoaraiosis	3.49	2.38–5.13	<0.0001	2.69	1.10–6.59	0.003

**Table 2:** Multivariable logistic regression model of significant variables including LA stratified according to Fazekas scale and using symptomatic patient belonging as dependent variable

Dependent variable: Symptomatic	OR	Nonadjusted	*p*	OR	Adjusted	*p*
Independent variable		95% CI			95% CI	
Age (years)	1.01	1.00–1.02	0.013	0.98	0.95–1.02	0.200
Previous Rankin scale	2.43	1.59–3.81	<0.0001	1.01	0.54–2.08	0.968
Ischemic heart disease	0.47	0.25–0.91	0.025	0.28	0.07–1.21	0.083
Previous stroke	1.18	1.02–1.96	0.005	1.02	0.65–1.98	0.107
Leukocytes × 10^3^/mmc	1.29	1.19–1.39	<0.0001	1.05	0.84–1.23	0.631
C‐reactive protein (mg/L)	1.40	1.23–1.59	<0.0001	1.20	0.99–1.50	0.088
Microalbuminuria (mg/24 h)	1.08	1.03–1.13	0.001	1.06	0.99–1.40	0.074
Leukoaraiosis grade				Ref		
No						
I				1.67	0.59–4.79	0.335
II				3.53	1.15–10.89	0.028
III				4.66	1.09–19.84	0.037

### LA association with sTWEAK

sTWEAK serum levels were analyzed in the entire sample. As detailed in Figure S1, symptomatic patients had significantly higher levels of circulating sTWEAK (7878.1 [5900.9–9604.7] pg/ml) compared to the asymptomatic patients (1940.3 [1067.4–3339.2] pg/ml) (*p* < 0.0001).

**Figure 1:**
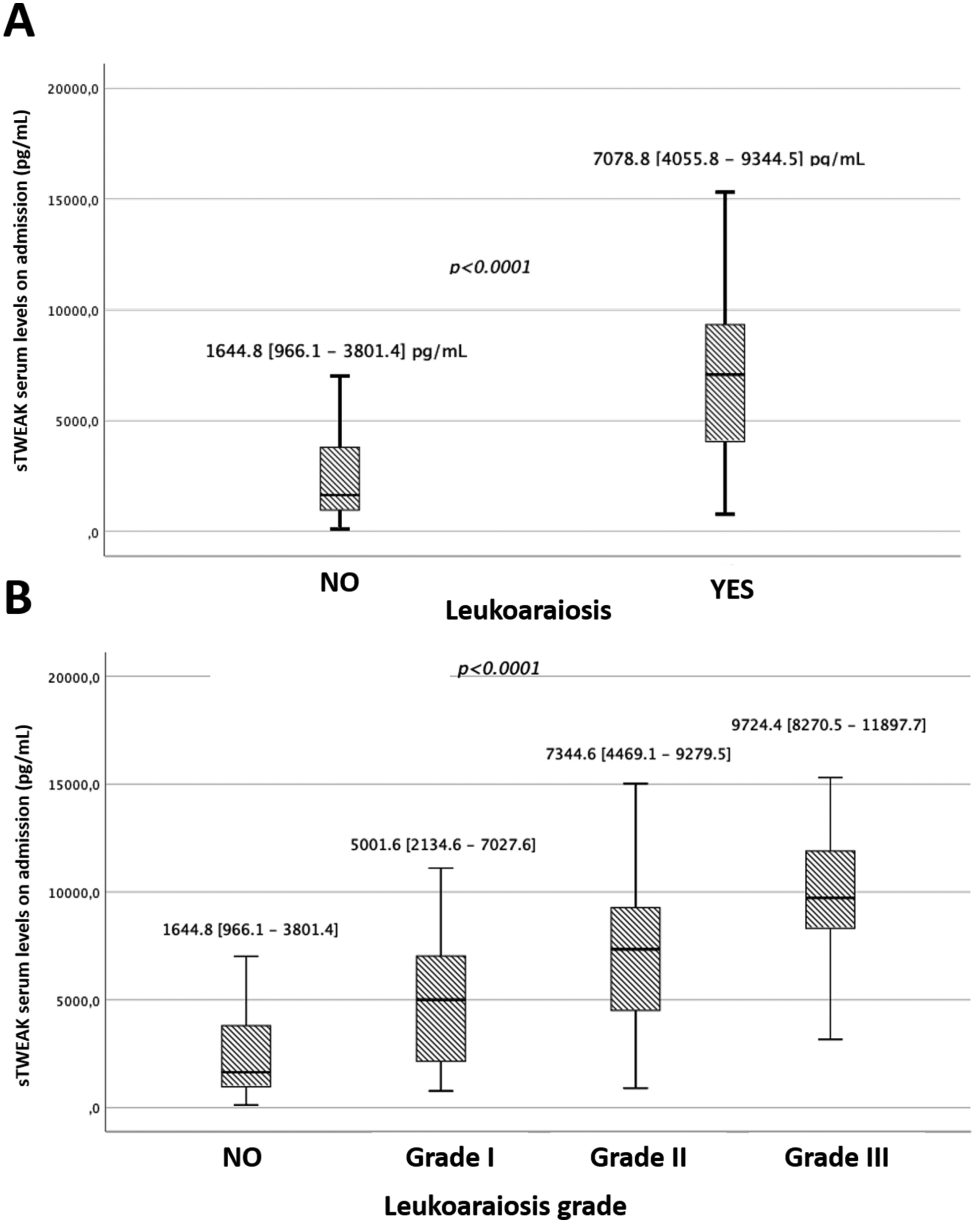
Serum sTWEAK levels in patients with and without LA (A) and in subjects stratified according to the degree of LA (B)

In addition, we analyzed the relationship of sTWEAK serum levels with the presence of LA in the whole sample as shown in Figure 1A. Patients with LA findings exhibited significantly (*p* < 0.0001) higher levels of sTWEAK (7078.8 [4055.8–9344.5] pg/ml) compared to patients without LA findings (1644.8 [966.1–3801.4] pg/ml). In order to relate sTWEAK serum levels with LA severity, subjects were stratified according to LA grade as measured by the Fazekas scale. As outlined in Figure 1B, patients who exhibited signs of LA showed significant differences (*p* < 0.0001) of sTWEAK, directly related to the increase in LA degree, evidencing the relationship between this molecule and LA severity.

**Figure 2:**
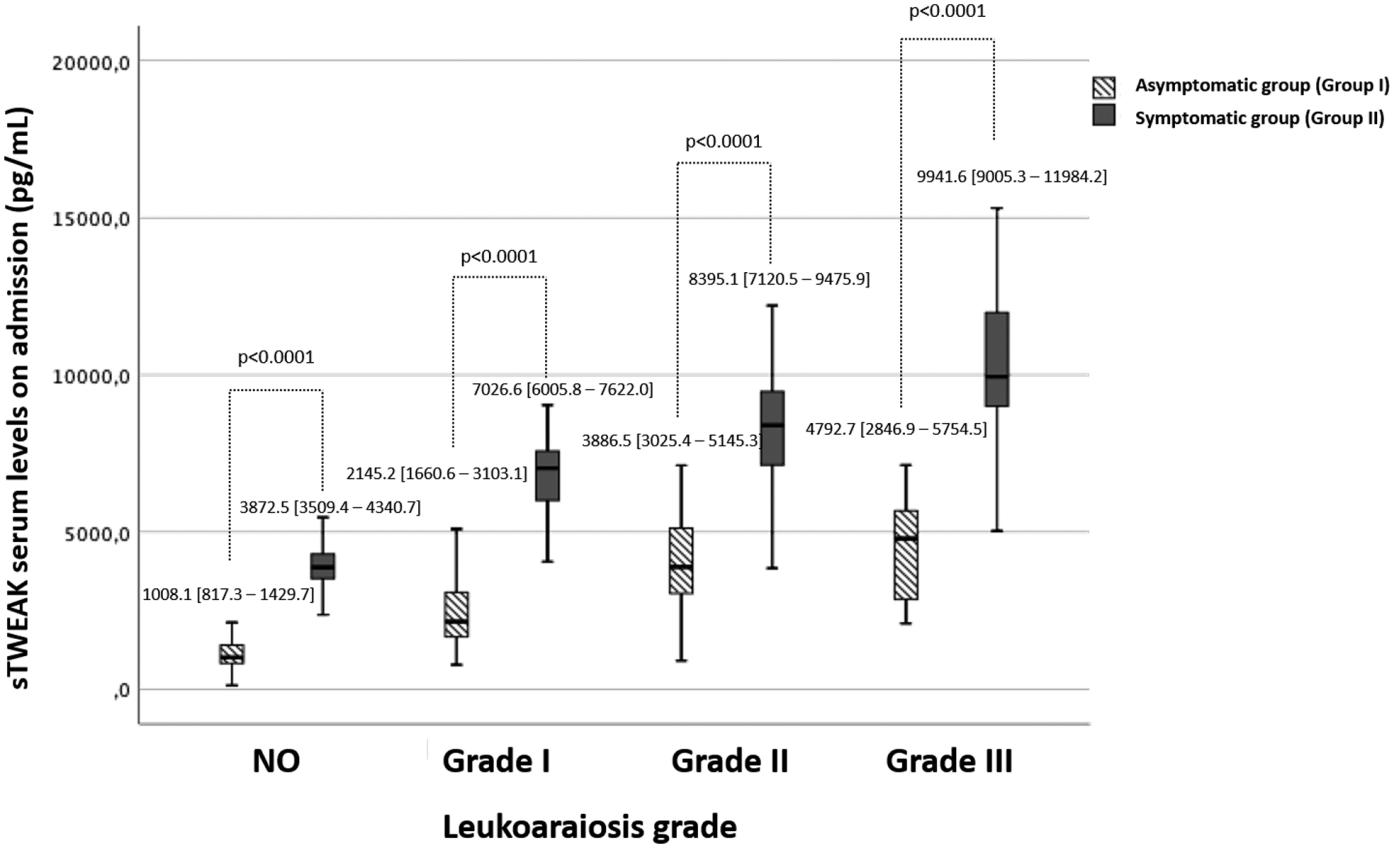
Serum sTWEAK levels in symptomatic and asymptomatic patients according to the degree of LA

To evaluate the relationship between the degree of LA and the role of clinical symptomatology, patients within each grade of LA were subdivided into nonsymptomatic subjects with high vascular risk (Group I) and symptomatic lacunar stroke or deep hemispheric ICH (Group II). The analysis showed an increase (*p* < 0.0001) in sTWEAK levels in the symptomatic group compared to the asymptomatic group in all LA grades (Figure 2). The trend in the increase in sTWEAK serum levels as a function of the increment of the degree of LA is maintained in both groups, being more prominent in patients with neurovascular symptomatology.

In line with these results, sTWEAK levels were included in a regression model using a neurovascular disease as dependent variable. As described in Table 3, sTWEAK levels are independently associated with the presentation of clinical symptoms (OR: 2.27, 95% CI: 1.70–3.04, *p* < 0.0001).

**Table 3:** Multivariable logistic regression model of significant variables including sTWEAK levels using symptomatic patient belonging as dependent variable

Dependent variable: Symptomatic	OR	Nonadjusted	*p*	OR	Adjusted	*p*
Independent variable		95% CI			95% CI	
Age (years)	1.01	1.00–1.02	0.013	0.96	0.92–1.01	0.105
Previous Rankin scale	2.43	1.59–3.81	<0.0001	1.04	0.44–2.48	0.924
Ischemic heart disease	0.47	0.25–0.91	0.025	0.07	0.05–1.64	0.219
Previous stroke	1.18	1.02–1.96	0.005	1.15	0.86–1.92	0.129
Leukocytes × 10^3^/mmc	1.29	1.19–1.39	<0.0001	1.05	0.80–1.39	0.712
C‐reactive protein (mg/L)	1.40	1.23–1.59	<0.0001	1.28	0.95–1.70	0.104
Microalbuminuria (mg/24 h)	1.08	1.03–1.13	0.001	1.07	0.97–1.17	0.074
sTWEAK (ng/ml)	3.64	2.19–5.90	<0.0001	2.27	1.70–3.04	0.167

In order to evaluate the relationship between LA and sTWEAK, both variables were included in the same multivariable regression model. In this case, LA lost its significance in the adjusted model (OR: 1.58, 95% CI: 0.93–7.35, *p* = 0.073) while sTWEAK ng/ml increased its relationship with neurovascular symptomatology (OR: 5.06, 95% CI: 2.66–9.75, *p* < 0.0001) as detailed in Table 4.

**Table 4:** Multivariable logistic regression model of significant variables including both LA and sTWEAK levels using symptomatic patient belonging as dependent variable

Dependent variable: Symptomatic	OR	Nonadjusted	*p*	OR	Adjusted	*p*
Independent variable		95% CI			95% CI	
Age (years)	1.01	1.00–1.02	0.013	0.97	0.92–1.03	0.280
Previous Rankin scale	2.43	1.59–3.81	<0.0001	1.02	0.29–2.35	0.715
Ischemic heart disease	0.47	0.25–0.91	0.025	0.41	0.02–2.13	0.468
Previous stroke	1.18	1.02–1.96	0.005	1.25	0.15–3.37	0.378
Leukocytes × 10^3^/mmc	1.29	1.19–1.39	<0.0001	1.34	0.95–1.89	0.089
C‐reactive protein (mg/L)	1.40	1.23–1.59	<0.0001	1.40	0.89–2.20	0.138
Microalbuminuria (mg/24 h)	1.08	1.03–1.13	0.001	1.11	0.97–1.27	0.132
Leukoaraiosis	3.49	2.38–5.13	<0.0001	1.58	0.93–7.35	0.073
sTWEAK (ng/ml)	3.64	2.19–5.90	<0.0001	5.06	2.66–9.75	<0.0001

## Discussion

The pathogenesis of LA is still unknown and not fully understood, yet it is a common denominator in various neurological disorders. LA has been extensively associated with vascular irregularities and more specifically with cSVD.^36^, ^37^, ^38^ Several clinical trials have established an association of LA with lacunar infarction,^39^ hemorrhagic stroke,^40^ and ischemic stroke,^7^ and previous literature show that patients with extensive LA are twice as likely to develop stroke. Also, LA showed its involvement in hemorrhagic transformation after reperfusion therapies.^15^, ^41^ Likewise, LA appears in asymptomatic subjects as a result of normal aging, which provides further evidence of the need to conduct an exhaustive LA characterization in order to obtain possible targets to stop its progression and prevent the appearance of other pathologies.^5^, ^8^, ^16^, ^18^

Currently, characterization by imaging techniques is limited and, in many cases, does not go beyond the use of the qualitative known scales.^42^, ^43^ They need to be combined with the new biomarkers in order to pinpoint the type of LA. One such potential markers and the focus of the present study is sTWEAK, a type II transmembrane glycoprotein of the TNF superfamily that binds to a transmembrane type I protein (Fn14). sTWEAK is involved in the expression levels of cytokines, cell adhesion molecules, tight junction proteins, and matrix metalloproteinases (MMPs) in cultured endothelial cells.^21^, ^35^, ^44^ In vitro, it also has been shown to alter the permeability of a blood‐brain barrier (BBB) model.^34^, ^35^, ^44^ In this regard, the possibility of blocking the activation of the sTWEAK‐Fn14 system could represent an important diagnostic and therapeutic option.^44^, ^45^

In this work, we conducted an observational study of a total of 624 patients divided into an asymptomatic and a symptomatic group. Compared with the asymptomatic subjects, symptomatic patients showed higher levels of inflammation markers such as C‐reactive protein and number of circulating leucocytes, as well as microalbuminuria, probably due to the presence of vascular pathologies,^46^, ^47^ that can be also noted in a higher previous modified Rankin scale detailed in the bivariate descriptive study. In this study, we first focused on the MRI analysis of LA present in the whole sample and the analysis of the grades according to the Fazekas scale. Patients who showed LA presence exhibited an association with neurological pathologies. Likewise, when this analysis was broken down according to the grade of LA, the ratio grew as the degree of LA is increased for grades II and III, but without reaching significance in grade I patients. Our analysis confirms one of the main objectives of this study: the association of LA and its severity with neurovascular pathologies, which is in line with previous studies.^7^, ^38^, ^39^, ^41^, ^47^ Furthermore, the lack of association of grade I LA strengthens the hypothesis of the causal implication of LA in the possible development of pathologies, and is a justification for the evaluation and detailed characterization of LA as a prognostic factor and therapeutic target to halt its advance.^48^

Although the involvement of the vascular component in the onset of the disease has gained strength, LA can currently be considered as a process of multiple origin, where protein expression patterns of the endothelium show a broad variability^8^, ^48^, ^49^ in response to different neurovascular environment.^8^, ^21^, ^33^, ^39^, ^45^ In this regard, the second aim of this work was the analysis of sTWEAK. Accordingly, our first step was to analyze the relationship between sTWEAK levels and clinical symptomatology. In line with similar works,^31^, ^50^ circulating sTWEAK levels were higher in our symptomatic patients compared to nonsymptomatic individuals. The pathogenic effect of this molecule has been previously reported in vascular pathologies, as sTWEAK disrupts the architecture of the BBB and induces the overexpression of MMP‐9 and pro‐inflammatory cytokines in the brain leading to endothelial dysfunction, pointing to a possible relationship with LA processes.^19^, ^33^ Once the symptomatic vascular association with sTWEAK was demonstrated, we proceeded to evaluate its relationship with LA. We observed that patients with LA not only had higher levels but when stratified by LA degree, sTWEAK levels increased as the severity of LA increased. Patients with grade III LA had almost six times more sTWEAK than those who did not show LA, which supports the notion that this molecule may play a role in the progression and development of LA. Furthermore, to better understand the relationship between sTWEAK and LA, we divided the sample according to the degree of LA and its assignment to the symptomatic and nonsymptomatic study groups. We observed that sTWEAK levels for both groups increased as the LA grade increased, but much more so in symptomatic patients who showed significantly higher levels in all LA grades. These differences were also maintained in symptomatic patients who did not show LA, although with lower levels. This confirms the crucial role of this molecule in vascular integrity as shown in similar studies.^32^, ^35^, ^44^ Blood collection times along pathology evolution seems to be an important point to take into account; similar works in patients with hypertension and diabetes showed different fluctuations in sTWEAK concentration.^51^, ^52^ The registered high levels of sTWEAK establish a double association between LA degree and neurovascular pathologies, heralding the potential of sTWEAK as an important target in the development of these diseases. In addition, when we compared the sTWEAK levels in symptomatic patients, patients with grade III LA showed 2.5‐fold higher sTWEAK levels than patients with no LA, which suggests that the generation of LA may not only be attributable to clinical symptomatology but causality may also be attributed to this molecule. This causality is also supported by the proposed regression models in which LA shows an association with neurovascular pathologies as well as sTWEAK. However, when they are included together in the same model, LA loses its significance while the relationship with sTWEAK increases. These results suggest that sTWEAK levels are associated with clinical manifestations through the development of LA. Nonetheless, our study showed some limitations. First, we conducted a retrospective single‐center study without preclinical data to support the molecular mechanisms involved in the interactions of sTWEAK with the different pathologies. Second, we studied sTWEAK and not the TWEAK‐Fn14 association, which could clarify the mechanism of action. In this regard, future studies of the pleiotropic metabolic pathways of sTWEAK involved in several diseases^53^ could help prospective studies focus on more than one biomarker. The strong points of this work are the unbiased screening of individuals, the high number of patients enrolled, and the focus on the assessment of a specific biomarker.

## Conclusions

LA showed a higher prevalence in patients with symptomatic lacunar stroke or deep hemispheric ICH. There is an association between sTWEAK levels and LA degree, so further studies are necessary to explore sTWEAK as therapeutic target to mitigate LA progression associated with cerebrovascular diseases.

## Author Contributions

AS‐C, JC, PH, and RI designed the study; AS‐C, AC, IL‐D, MR‐Y, and TS acquired clinical data for further analysis; PA and MA‐A elaborated tables and designed images; AS‐C, TS, FC, and JC analyzed the collected data and performed statistical analysis; AS‐C, JMP, and PH drafted the manuscript.

## Conflict of Interest

The authors report no conflict of interest.

## Supporting information


**Table S1.** Bivariate analysis of demographic, molecular, and neuroimaging variables for asymptomatic and symptomatic groups.
**Figure S1.** Serum levels of sTWEAK on admission in asymptomatic and symptomatic patients.
