# Supplementary material for: sTWEAK is a leukoaraiosis biomarker associated with neurovascular angiopathy
Source: Ann Clin Transl Neurol. 2022 Jan 21;9(2):171–80. doi: 10.1002/acn3.51502 (PMC8862435; doi:10.1002/acn3.51502)
Supplement: Supplementary file 1 — Table S1. Bivariate analysis of demographic, molecular, and neuroimaging variables for asymptomatic and symptomatic groups. Figure S1. Serum levels of sTWEAK on admission in asymptomatic and symptomatic patients. [file ACN3-9-171-s001.docx]

***sTWEAK is a leukoaraiosis biomarker associated to neurovascular angiopathy.***

Supplementary Material

**Supplemental Table 1.** Bivariate analysis of demographic, molecular and neuroimaging variables for Asymptomatic and Symptomatic groups

|  |  |  |  |  |
| --- | --- | --- | --- | --- |
|  |  | **Asymptomatic**  **n = 242** | **Symptomatic**  **n = 382** | ***p*** |
| Demographic variables |  |  |  |  |
| Age, years |  | 70.1 ± 9.6 | 71.5 ± 12.7 | 0.017 |
| Female gender, % |  | 47.5 | 47.4 | 0.519 |
| Previous Rakin sacale |  | 0 [0, 0] | 0 [0, 1] | <0.0001 |
| Arterial hypertension, % |  | 81.8 | 84.0 | 0.510 |
| Diabetes, % |  | 26.0 | 26.7 | 0.926 |
| Smoking, % |  | 18.6 | 20.2 | 0.679 |
| Alcohol consumption, % |  | 10.3 | 12.3 | 0.521 |
| Hyperlipidemia, % |  | 44.2 | 37.2 | 0.093 |
| Peripheral arterial disease, % |  | 1.6 | 2.9 | 0.169 |
| Ischemic heart disease, % |  | 2.4 | 10.3 | 0.027 |
| Atrial fibrillation, % |  | 1.5 | 4.7 | 0.120 |
| Heart failure, % |  | 1.2 | 2.4 | 0.102 |
| Carotid disease, % |  | 1.3 | 2.9 | 0.253 |
| Previous stroke, % |  | - | 7.1 | 0.019 |
| Molecular and clinical variables |  |  |  |  |
| Hemoglobin, g/L |  | 14.2 ± 1.7 | 13.1 ± 2.1 | 0.619 |
| Leukocytes x 10^3^/mmc |  | 7.3 ± 1.9 | 8.8 ± 2.9 | <0.0001 |
| Platelets, x10^3^/mL |  | 224.7 ± 96.1 | 209.1 ± 78.2 | 0.958 |
| Glycemia, mg/dL |  | 118.9 ± 39.5 | 135.0 ± 53.9 | 0.056 |
| Glycosilated hemoglobin, % |  | 5.9 ± 0.9 | 6.1 ± 1.2 | 0.076 |
| Fibrinogen, mg/dL |  | 401.2 ± 78.0 | 448.3 ± 91.3 | 0.079 |
| C reactive protein, mg/L |  | 1.29 ± 1.87 | 4.51 ± 5.17 | <0.0001 |
| Microalbuminuria, mg/24 h |  | 3.4 ± 7.2 | 13.3 ± 18.1 | <0.0001 |
| LDL-cholesterol, mg/dL |  | 115.7 ± 35.8 | 111.1 ± 37.7 | 0.520 |
| HDL-cholesterol, mg/dL |  | 42.1 ± 14.0 | 41.4 ± 17.5 | 0.549 |
| Triglycerides, mg/dL |  | 125.6 ± 60.2 | 120.5 ± 65.4 | 0.924 |
| Intima-media thickness, mm |  | 0.9 ± 0.2 | 0.9 ± 0.2 | 0.329 |
| Neuroimaging variables |  |  |  |  |
| Degree of leukoaraiosis, % |  | 62.0 | 85.1 | <0.0001 |
| No |  |  |  | <0.0001 |
| Grade I |  | 38.0 | 14.9 |  |
| Grade II |  | 36.4 | 24.9 |  |
| Grade III |  | 19.8 | 28.8 |  |


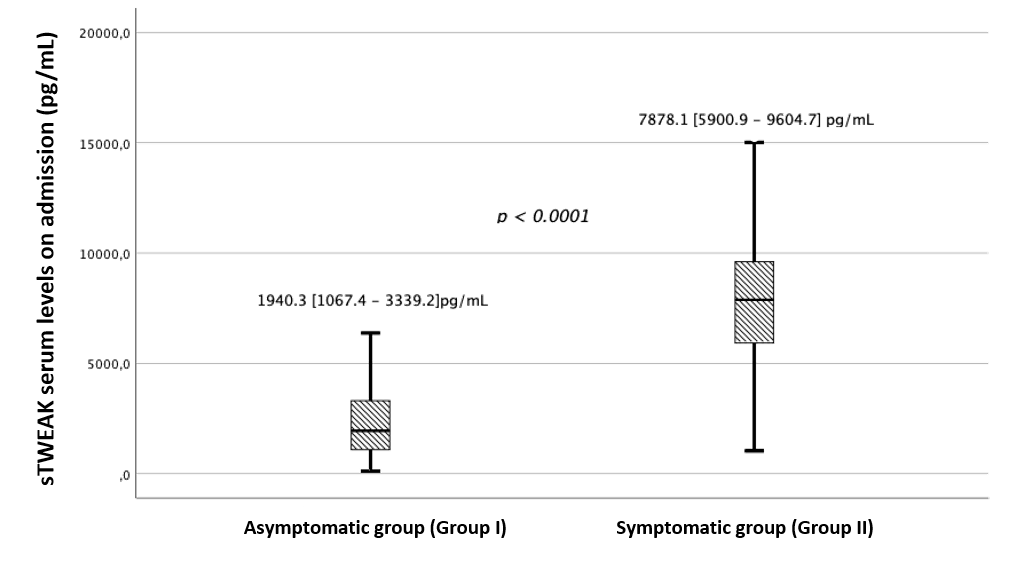


**Supplemental Figure 1.** Serum levels of sTWEAK on admission in asymptomatic and symptomatic patients
